# A Retrospective Study on the Avoidability of Ventriculoperitoneal Shunt Infections in a University Hospital in Al-Khobar, Saudi Arabia

**DOI:** 10.7759/cureus.13135

**Published:** 2021-02-04

**Authors:** Maan A Albehair, Mazen A Alosail, Naif M Albulwi, Ahmed AlAssiry, Fahad A Alzahrani, Ammar Bukhamsin, Ahmed Ammar

**Affiliations:** 1 Medicine, Imam Abdulrahman Bin Faisal University, Dammam, SAU; 2 Neurosurgery, King Fahd Hospital of the University, Khobar, SAU

**Keywords:** ventriculoperitoneal shunt, infections, cerebrospinal fluid

## Abstract

Ventriculoperitoneal shunt infection is a major complication and the main cause of shunt failure, contributing to a high rate of morbidity and mortality among patients requiring prolonged hospitalization. Shunt infection-related complications are considered a global burden of hydrocephalus worldwide. In our hospital, King Fahad Hospital of the University, the rate of infections in similar cases reached 8% during the period from 1999 to 2001; an increase in this rate was observed in the past two years.

This study analyzed the infections that occur after ventriculoperitoneal shunt placement in patients with hydrocephalus and related conditions during the period from January 2012 to April 2017. The objectives of this study were to analyze the rate of cerebrospinal fluid infections in different age groups at the King Fahd Hospital of the University and to identify the causative pathogens and methods of reducing the rate and consequences of such infections. In this retrospective study, the electronic medical records of 266 patients were reviewed and those of 131 patients were included and analyzed. We found that the prevalence rate of shunt-related infections was 24.4%, which indicates the importance of this problem. Staphylococcus epidermidis is the most commonly implicated microorganism. The most affected age groups were those of preschool children and infants. Individuals who were older than 74 years were the least affected. It was found that delayed infections were the most common type of VP shunt infection among the study population and there was no difference in the most common organisms between early, delayed, and late infections. The mean duration of antibiotic treatment used was 19.76 days. In conclusion, postoperative infections are significantly common in patients who undergo shunt-related surgeries.

## Introduction

A ventriculoperitoneal (VP) shunt is a medical apparatus that is utilized to relieve elevated intracranial pressure. There is a possibility that infections may be developed and spread locally from one site to another because of the disturbance created by surgical operations creating a possible scenario of a high infection rate. The symptoms of postoperative infections may be subtle or mild thereby patients often acclimatize with them and fail to seek medical attention such that the healthcare professionals can diagnose it and take the appropriate steps to manage it. Operations involving the parts of the body with less normal organisms result in the lowest number of postoperative infections. The infections are also shown to be significantly associated with wound dehiscence. There are some indications for VP shunt including congenital hydrocephalus, tumor leading to cerebrospinal fluid (CSF) blockage of the lateral or third ventricles (the posterior fossa and intraspinal tumors), and posthemorrhagic hydrocephalus. Hence, we will identify the difference between organisms that present as early, delayed, or late infections of VP shunt. We would like to clarify the differences between these organisms and their types and durations of antibiotics [1].

## Materials and methods

Purpose

The purpose of this retrospective study was to assess if VP shunt infections are avoidable based on the incidence rate and causative organism in King Fahd Hospital of the University (KFHU). And to discuss methods of reducing the rate and consequences of such infections in the KFHU in Al-Khobar, Kingdom of Saudi Arabia, during the period from January 2012 to April 2017.

Methods

Hospital Setting

The study was conducted at the KFHU in Al-Khobar, Kingdom of Saudi Arabia. It has a capacity of 500 beds, which are distributed in a four-floor building. The hospital is a secondary medical and healthcare provider in Khobar city, which has a population of about 200,000. It services a wide population of patients coming from all over the Eastern Region, which is considered the third-most-populous region in Saudi Arabia.

Between 2012 and 2017, about 131 cases of hydrocephalus were managed in the hospital through the insertion of VP shunts.

Study Population

All episodes of infections that occurred from January 2012 to April 2017 in patients who underwent VP shunting at the facility were included. The criteria for concluding that an infection is related to the insertion of a VP shunt are as follows: (i) growth of relevant pathogens in the CSF, VP shunt tip, and surgical wound; (ii) fever of >38 °C, headache, and neurologic symptoms and signs. The criterion for the selection of the subjects was a first-time diagnosis of a condition that required management using a VP shunt. The selected patients were categorized according to their medical condition, the type of procedure conducted, and the causative organism of infection from CSF culture through a lumbar puncture or direct puncture of the shunt reservoir. The following patients were excluded from this study: patients who had their shunts removed before the diagnosis of an infection, those who were diagnosed with a shunt-related infection in a different health facility, and those whose condition was managed by performing a CSF diversion procedure rather than by VP shunting.

Ethics

The study proposal was reviewed and approved by the institutional review board (IRB) at Imam Abdulrahman Bin Faisal University, Dammam, Saudi Arabia, vide IRB number (IRB-UGS-2019-01-370).

Data Collection

From November 2019 to February 2020, data were collected by a retrospective review of electronic charts. Data on age, sex, the procedure used for the management of the medical condition, the manifestations of infections, and the use of antimicrobial agents were collected. The used devices for the identification of the causative organisms are matrix-assisted laser desorption ionization-time of flight mass spectrometry (MALDI-TOF) (aka Vitek-ms, bioMérieux, Marcy-l'Étoile, France) and Vitek2 if not identified by Vitek-ms. The infections were categorized as early (less than a month after the shunt surgery), delayed (1-12 months), or late (>1 year).

Follow-up and treatment outcomes

The outcomes of treatment were evaluated by reviewing subsequent hospital admissions and contacting the healthcare professionals who managed the patients. In this study, relapse or treatment failure was defined by the occurrence of infection within three months after remission of the symptoms since the shunt was placed as well as by the isolation of the same pathogen. A new shunt-related infection was defined by the manifestation of an infection more than three months after VP shunting and by the isolation of a different organism from the one found at the time of CSF diversion. In a review between the years 2012 and 2017, about 131 cases of hydrocephalus were managed in the hospital through the insertion of VP shunts, and they were evaluated in regards to the outcomes of treatment and follow-up.

Statistical analysis and presentation

All calculations were performed using the Statistical Package for the Social Sciences (SPSS 22.0; IBM Corp, Armonk, NY). Microsoft Excel (Microsoft Corporation, Redmond, WA) spreadsheets were used to illustrate figures. Further analysis was performed by computation and summing and converting the figures in percentage form to give a clear picture of the trend and observation, which would allow easy comparison. The analyzed data are presented in the form of tables and pie charts to illustrate the relationships between all the variables of the study.

## Results

Among the 266 patients who were initially included in the study, data obtained from the medical records of 131 patients were used for conducting analyses. The remaining 135 patients were excluded according to the exclusion criteria. Among the 131 patients, 32 showed positive culture results. The data that were used for analyses were obtained from patient records such as readmission notes or charts showing the development of any complications.

**Figure 1:**
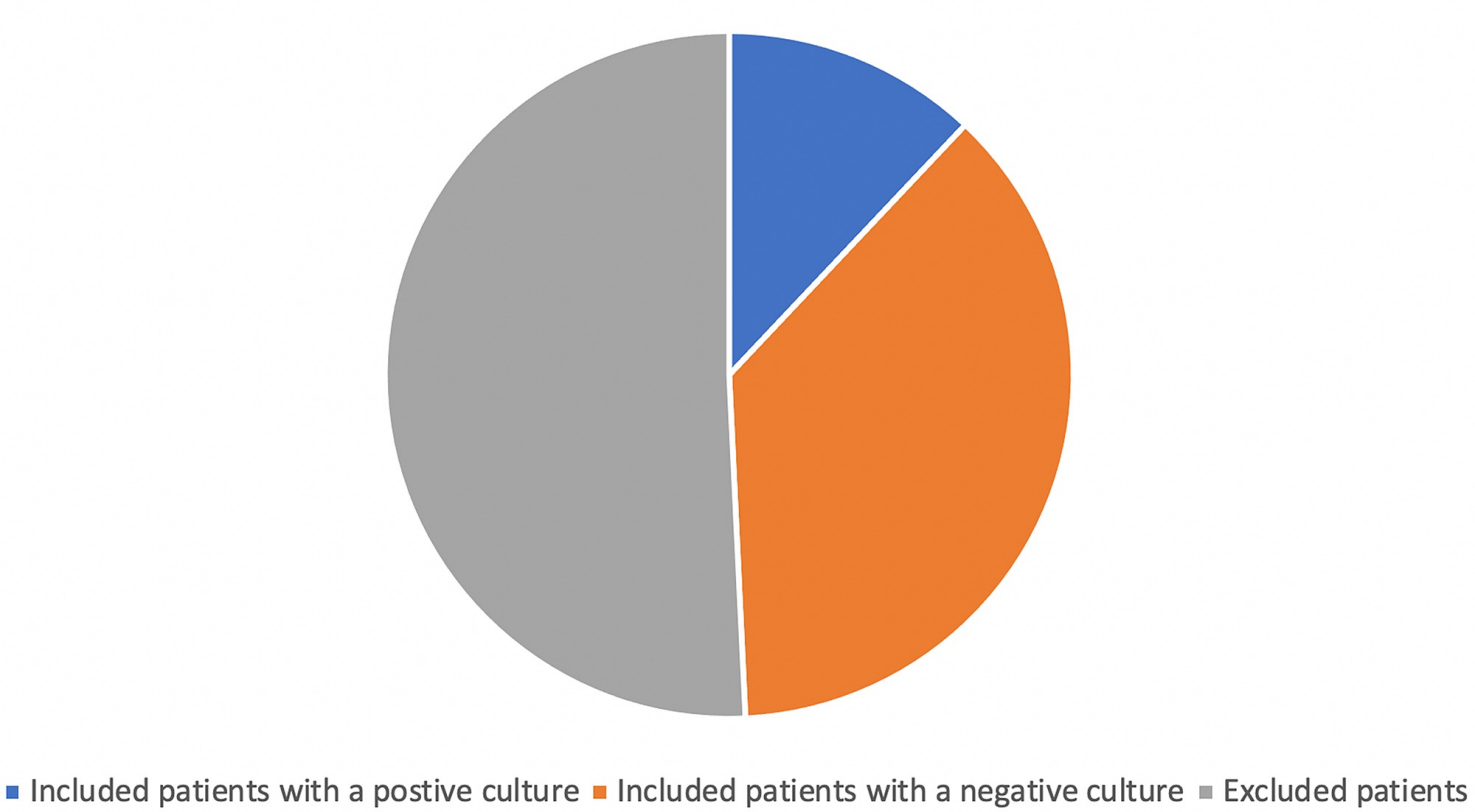
Total number of related neurological operations

The patients who showed positive CSF culture results accounted for 24.4% of the total patients. Figure 1 shows the total number of related neurological operations conducted and illustrates the high rate of infection in the included patients.

Postoperative infections were managed by placing an external ventricular drain (EVD) and by the administration of antibiotics until the clearance of the infection. The rate of infections among patients who underwent VP shunting was 24.4% (32/131). The overall postprocedural infection rate was 24.4%. The age-based rates of postoperative infections were as follows: neonates, 3.12%; infants, 25%; preschool children (1-5 years), 37.5%; schoolgoing children (6-12 years), 12.5%; adolescents (13-18 years), 12.5 %; adults (19-64 years), 9.37%; and elderly individuals (>64 years), 0%. This data is presented in Table 1.

**Table 1:** Shunt infection according to the age of the patient

Age Group	Number/32	Percentage (%)
Neonate (<1 month)	1	3.12%
Infant (1–11 months)	8	25%
Preschool (1–5 yrs)	12	37.5%
Schoolgoing children (6–12 yrs)	4	12.5%
Adolescent (13–18 yrs)	4	12.5%
Adults (19–64)	3	9.37%
Elderly (>65 yrs)	0	0%

From those 32 patients who showed infectious organisms in their culture, 10/32 were early infection and 8/32 were late while delayed infections happened to 12/32, and in 2/32, the time of infection was unknown.

VP shunting is the most common surgery conducted for the management of hydrocephalus at KFHU. It should be noted that the absolute number of infected cases for each of the surgical techniques should not be used for determining the procedures with the highest rates of infection. Instead, the percentage of infected patients out of the total number of patients who underwent such procedures should guide an accurate and substantial comparison between the postoperative infections linked to the respective surgical techniques. Furthermore, these statistical findings consider the fact that VP shunting was one of the multiple surgeries undergone by some patients, either for the management of a complication or as a concomitant procedure.

The most common causative agent was Staphylococcus epidermidis, which accounted for 71.8% (23/32) of all infections (Table 2). The least implicated microorganisms in the post-VP shunting infections were common infective agents such as Pseudomonas aeruginosa, Acinetobacter baumannii, Escherichia coli, Staphylococcus capitis, Streptococcus group B, Enterococcus, Klebsiella oxytoca, Pseudomonas sp., Enterococcus gallinarum, and Staphylococcus hominis. The most common condition that required VP shunting was congenital hydrocephalus (communicating and obstructive type), and patients who underwent VP shunting for the management of this condition accounted for 27.2% of the infection rate, as shown in Table 3. Moreover, 6/10 early infections were caused by Staphylococcus epidermis while 7/8 late complications were caused by Staphylococcus epidermis, and 9/12 of delayed infections were caused by Staphylococcus epidermis in addition to other infectious agents species. The mean duration of antibiotic treatment was 19.76 days. Table 4 shows patient-wise data on age, sex, and causative organism.

**Table 2:** List of causative organisms

Name of organism	Number of patients	(%)
Staphylococcus epidermidis	23	71.8%
Enterobacter cloacae	5	15.6%
Enterococcus faecalis	2	6.2%
Staphylococcus aureus	2	6.2%
Acinetobacter baumannii	1	3.2%
Pseudomonas aeruginosa	1	3.2%
Escherichia coli	1	3.2%
Staphylococcus capitis	1	3.2%
Klebsiella oxytoca	1	3.2%
Pseudomonas species	1	3.2%
Enterococcus gallinarum	1	3.2%
Staphylococcus hominis	1	3.2%
Streptococcus group B	1	3.2%
Enterococcus	1	3.2%

**Table 3:** Diagnosis at admission and postprocedural infection rate

Diagnosis	Number of infection/procedures
Congenital hydrocephalus	18/66 (27.2%)
Spina bifida with hydrocephalus	10/31 (32.2%)
Obstructive hydrocephalus secondary to space-occupying lesion	1/10 (10%)
Dander Walker syndrome + hydrocephalus	2/9 (22.2)
Hydrocephalus secondary to vascular etiology	0/5 (0%)
Normal-pressure hydrocephalus	0/3 (0%)
Arachnoid cyst + hydrocephalus	0/2 (33%)
Post-traumatic hydrocephalus	0/2 (16%)
Intraventricular hemorrhage of prematurity	1/1(100%)
Colloid cyst with hydrocephalus	0/1 (0%)
Post-infectious hydrocephalus	0/1 (0%)

**Table 4:** Patient-wise data on age, sex, and causative organism

#	Age	Sex	Organism
1	27 years	Female	Staphylococcus epidermidis
2	24 years	Male	Enterobacter cloacae, Klebsiella oxytoca
3	15 years	Male	Staphylococcus epidermidis
4	15 years	Female	Staphylococcus epidermidis, Streptococcus group B, Enterococcus
5	13 years	Female	Escherichia coli
6	14 years	Female	Staphylococcus epidermidis
7	10 years	Male	Staphylococcus epidermidis
8	7 years	Female	Staphylococcus epidermidis
9	8 years	Female	Staphylococcus epidermidis
10	5 years	Female	Staphylococcus epidermidis, Enterococcus faecalis
11	5 years	Male	Staphylococcus epidermidis, Acinetobacter baumannii
12	4 years	Female	Staphylococcus epidermidis
13	4 years	Male	Staphylococcus epidermidis, Pseudomonas species
14	4 years	Male	Enterococcus gallinarum
15	2 years	Female	Staphylococcus aureus
16	3 years	Male	Staphylococcus hominis, Staphylococcus epidermidis
17	6 years	Male	Enterobacter cloacae
18	30 years	Male	Enterobacter cloacae
19	4 years	Female	Staphylococcus epidermidis
20	5 months	Female	Staphylococcus epidermidis
21	59 days	Female	Staphylococcus epidermidis
22	14 days	Male	Staphylococcus epidermidis
23	20 months	Male	Staphylococcus epidermidis
24	14 months	Female	Staphylococcus epidermidis
25	2 months	Male	Staphylococcus epidermidis
26	2	Male	Staphylococcus epidermidis, Enterobacter cloacae
27	2 months	Male	Pseudomonas aeruginosa
28	16 months	Female	Staphylococcus epidermidis
29	50 days	Female	Staphylococcus epidermidis
30	4 months	Female	Staphylococcus epidermidis, Enterococcus faecalis, Staphylococcus capitis
31	5 months	Female	Enterobacter cloacae
32	3 months	Male	Staphylococcus aureus

## Discussion

VP shunts are of major concern after surgery involving the ventricles, and they are the most common surgeries conducted at KFHU for the management of hydrocephalus. This retrospective study thus justifies this statement due to the high prevalence of this condition, with the 24.4% (31/131 patients) infection rate linked to the respective surgical techniques, taking into consideration that preschoolers have the highest percentage among others. Staphylococcus epidermidis is the most common causative agent since it is a normal skin flora, accounting for 71.8% (23/32 patients) of the total cases. The most common condition was congenital hydrocephalus (communicating and obstructive type), which accounted for the 27.2% infection rate of the total patients with congenital hydrocephalus who underwent VPS. Furthermore, as a difference in causative organisms between early, late, and delayed, 6/10 of early infections were caused by Staphylococcus epidermis and 7/8 late complications were caused by Staphylococcus epidermis while 9/12 of delayed infections were caused by Staphylococcus epidermis in addition to other infectious agents species.

However, the novelty of this study is that there is no difference between early and late VPS infection in causative organisms while in delayed infection, few cases showed a difference, and other causative organisms were detected.

As a comparison with other studies, VP shunting is associated with a higher number of infections than are other procedures because of the presence of a large number of normal flora in the peritoneum that can be pathogenic to other tissues of the body such as CSF [2-3]. However, VP shunting is the second most common approach used for the management of hydrocephalus at KFHU. The protocol used for the management of hydrocephalus at the facility was affected by several factors.

The protocol observation shows that the efficacy of the techniques plays an important role in influencing the result of techniques commonly practiced at the facility. KFHU depends on evidence-based practice (EBP), which involves the integration of research-proven practices in the management protocols. Therefore, a conclusion is that the underlying cause for the observation of the different rates of utilizing the techniques in hydrocephalus management at a facility such as VPS is possible. Further, the trend influences the occurrences of postoperative infections in the patients who undergo such procedures in the theater. According to some international studies, Staphylococcus epidermidis, a normal skin flora, was the most commonly detected organism; however, a wide variety of other rare organisms have been detected in the current study (Table 2) [4-6].

Staphylococcus spp. mostly occur on the skin and superficial areas, according to a study that noted that infections caused by such agents are hard to avoid. This explains the observation as to why surgical incisions are prone to infections, which is why, after culture and sensitivity tests, the Staphylococcal bacteria are often isolated [7].

Furthermore, the fact that minimally invasive procedures predispose an individual to the lowest risk of postprocedural infection supports the observation that Staphylococcus poses the threat of surgical site infections. A surgical incision made through the skin to the ventricles and the peritoneum during VP shunting is thus a possible mechanism of transfer of the microorganisms to new sites, resulting in infections that manifest as postoperative complications. According to one study, a postprocedural infection was observed in 11 out of 142 patients (7.7%) who underwent ventricular access device placement: Staphylococcus epidermidis, four patients; Enterococcus faecalis, two patients; Escherichia, two patients; Pseudomonas aeruginosa, two patients; and Enterobacter cloacae, one patient [2].

CSF diversion measures are used in the setting of hydrocephalus, either congenital or secondary to different etiologies. It is used to divert CSF from the intraventricular compartment to another compartment for absorption or externalized for CSF blood/infection clearance, in addition to endoscopic third ventriculostomy, which is considered superior in the concept of not carrying foreign shunt hardware [8]. Surgery-related infections have been shown to be caused by several factors such as hematogenous spread and contact with infected equipment; furthermore, these infections could be airborne. Placement of a foreign shunt in the body, therefore, significantly contributes to the development of CSF infections in patients undergoing VP shunting. Therefore, only sterilized hardware must be inserted into the body. Various preventive measures and precautions are carried out to reduce the rate of CSF infection. Prophylactic measures, including maintenance of patient/physician hygiene, decreasing the number of persons present in the operative room, short operative time, and proper instrument sterilization, must be employed to reduce the rate of CSF infection. Thus, to reduce the hospital shunt infection rate, KFHU implemented a strict shunt protocol in April 2017 that should be followed and attached to every patient’s documents; this protocol was adapted and modified from various previous studies [9]. Its employment led to a significant decrease in the shunt infection rate (approximately 1%). However, scientific validation of this protocol using a large study population is necessary. The rate of infection determined in the current study indicates the virulence of the causative organisms.

A study noted that the virulence of a pathogenic organism plays a significant role in determining its ability and behavior in causing infections after surgery [10]. Such a note is shown by the difference in the most common microorganisms that cause infection in this regard.

In addition, patient-dependent factors affect the possibility of post-VP shunting infection. First, high levels of hygiene are associated with low levels of resistance against some infectious agents with low virulence. The same last-mentioned study reported that places in which this trend is seen are often societal with marked economic development which also translates to a higher level of healthcare [10]. The study, thus, provides examples of North American, European, and some Middle Eastern countries such as the Kingdom of Saudi Arabia. In this regard, low levels of resistance against some bacterial agents support the observation made in the infection rates observed in KFHU. This explains the significantly high levels of infection caused by organisms showing very low virulence such as Enterobacter aerogenes, Burkholderia cepacia, and Providencia stuartii. Such a trend may not be observed in populations showing high levels of tolerance against infectious agents implicated in the development of post-surgical infections. Therefore, the hygiene hypothesis may be closely linked to the issue of immune deficiency although it is not caused by a reduction in an individual’s immune status and socioeconomic status of the person. Second, the environmental exposure and nutritional status may affect the possibility of post-VP shunting infection.

A study conducted on post-shunting infection in a rural Kenyan population found that the majority of patients were aged less than six months and that a high percentage of drug-resistant gram-negative organisms was associated with a poor outcome [6]. The rate of infection was highest in adults, followed by preschool children. Such an observation agrees with some findings that have attributed certain factors to the development of postoperative infections. They include patient age, immune status, environmental exposure, and nutritional status.

In this regard, environmental exposure is defined by the exposure to microorganisms that can cause a disease or an infection. It encompasses factors such as hygiene and wound care by the patient or the nursing staff.

This explains the data presented in Table 1, that is, children and elderly individuals are more often affected by the least virulent organisms than are individuals of the remaining age groups. On the other hand, adults are mostly affected by organisms showing high virulence. Nutritional status indirectly affects immune status because the nutrients and minerals consumed by an individual are used for generating immune cells, which counteract the infections. Environmental exposure is defined as the presence of the infective organisms on materials and their transmission from infected individuals to healthy individuals via the various modes, which include airborne, waterborne, and direct contact. On the contrary, preschool children throughout adulthood showed a significantly higher postoperative infection rate because of the high level of environmental exposure at school, work, and other social gatherings.

As for other factors, one particular study reported that risk factors of infection included frequent revisions of the shunt, young age of patients, and causes of hydrocephalus [11]. These rates can be affected by other factors such as the duration of hospital stay, surgeon experience, surgical technique, procedural duration, and handling of the indwelling device during surgery.

The study contributes to the field of neurosurgery, specifically VP shunt operations where VP shunt infections can occur, leading to serious complications. Furthermore, the result of such a study should raise awareness regarding this issue among physicians who would be part of any VP shunt operation either preoperatively to prevent the infections and their complications or postoperatively to observe the patients and manage accordingly if such an issue present. Our study recommends the application of a shunt protocol to address perioperative pitfalls that are essential for reducing overall mortality and morbidity. The analysis of a common and wide variety of organisms mandates further analyzing the risk factors associated with the occurrence of infection, including patient conditions, hospital environment, operative theater settings, the etiology of hydrocephalus, repeated shunt revisions, prematurity of newborns, presence of concomitant diseases, prolonged hospital stay, surgeon's experience, and intraoperative overhandling of the shunt. The obtained evidence may be useful for the development of a protocol that can help reduce the rate of infections, especially those caused by the most common organisms, by reducing the duration of the surgery, maintaining an antiseptic technique, and using the double gloves technique. Further, there is a need to investigate any possible factor explaining the observed trend of postoperative infection in the different age groups. The findings of such a study may help control infections in these individuals, reduce the prevalence of post-shunt infections in all patients who undergo shunt-related and other surgeries significantly, and reduce mortality and morbidity rates.

Limitations

Due to the coronavirus disease 2019 (COVID-19) or severe acute respiratory syndrome coronavirus 2 (SARS-COV-2) pandemic crisis and the restriction on all hospitals, obtaining the last two years' data was difficult because a new protocol was applied at the hospital where the quality department at the hospital recorded two consecutive years with a zero rate of VP shunt infection. In addition, more information on current data like hospital stay length was not taken due to the restriction on hospital archives, as the data should be collected from the archives department. Also, there was a need for a biostatistical analysis specialist to analyze the data more, giving that analyzing the data, in general, was difficult due to the suspension of university work during this crisis for which we need the help of a biostatistical analysis specialist. Finally, more details of the outcomes of the study population were not investigated further.

## Conclusions

Postoperative infection is the major concern after VP shunting and is the most common surgery conducted at KFHU for the management of hydrocephalus. This retrospective study showed that the postoperative infection rate is high after VP shunting and that this infection rate is linked to the surgical techniques used and the age group of patients. Furthermore, Staphylococcus epidermidis was found to be the most common causative agent and congenital hydrocephalus was found to be the condition associated with the highest infection rate. Moreover, it was found that there’s no difference between early, delayed, and late VP shunt infection in regards to the most common causative organism. Future studies analyzing the risk factors of patients, hospital environment, and operative theater settings associated with the incidence of infection are necessary. Applying a shunt protocol addressing perioperative pitfalls is important to reduce overall mortality and morbidity. The etiology of hydrocephalus, repeated shunt revisions, prematurity of newborns, presence of concomitant diseases, prolonged hospital stay, surgeon's experience, and intraoperative overhandling of the shunt are considerable risk factors.
